# Linear low-dose extrapolation for noncancer health effects is the exception, not the rule

**DOI:** 10.3109/10408444.2010.536524

**Published:** 2011-01-13

**Authors:** Lorenz R Rhomberg, Julie E Goodman, Lynne T Haber, Michael Dourson, Melvin E Andersen, James E Klaunig, Bette Meek, Paul S Price, Roger O McClellan, Samuel M Cohen

**Affiliations:** 1 Gradient, Cambridge, Massachusetts, USA; 2 Toxicology Excellence for Risk Assessment (TERA), Cincinnati, Ohio, USA; 3 The Hamner Institutes for Health Sciences, Research Triangle Park, North Carolina, USA; 4 Department of Environmental Health, Indiana University, Bloomington, Indiana, USA; 5 Institute of Population Health, University of Ottawa, Ottawa, Ontario, Canada; 6 The Dow Chemical Company, Midland, Michigan, USA; 7 Advisor, Toxicology and Human Health Risk Analysis, Albuquerque, New Mexico, USA; 8 Department of Pathology and Microbiology, University of Nebraska Medical Center, Omaha, Nebraska, USA

**Keywords:** Additivity to background, dose-response, exposure measurement error, linear, nonlinear, population heterogeneity, threshold

## Abstract

The nature of the exposure-response relationship has a profound influence on risk analyses. Several arguments have been proffered as to why all exposure-response relationships for both cancer and noncarcinogenic end-points should be assumed to be linear at low doses. We focused on three arguments that have been put forth for noncarcinogens. First, the general “additivity-to-background” argument proposes that if an agent enhances an already existing disease-causing process, then even small exposures increase disease incidence in a linear manner. This only holds if it is related to a specific mode of action that has nonuniversal properties—properties that would not be expected for most noncancer effects. Second, the “heterogeneity in the population” argument states that variations in sensitivity among members ofthe target population tend to “flatten out and linearize” the exposure-response curve, but this actually only tends to broaden, not linearize, the dose-response relationship. Third, it has been argued that a review of epidemiological evidence shows linear or no-threshold effects at low exposures in humans, despite nonlinear exposure-response in the experimental dose range in animal testing for similar endpoints. It is more likely that this is attributable to exposure measurement error rather than a true non-threshold association. Assuming that every chemical is toxic at high exposures and linear at low exposures does not comport to modern-day scientific knowledge of biology. There is no compelling evidence-based justification for a general low-exposure linearity; rather, case-specific mechanistic arguments are needed.

## Contents

Abstract..........1
Introduction..........2
Framing the problem..........3
Additivity to background..........5
The no-threshold proposal for noncancer toxicity is at variance with decades of experience in observing exposureresponse relationships in pharmacology and toxicology, both within and below the usual experimental range for environmental chemicals..........9
The no-threshold proposal is at variance with basic tenets of homeostasis—the robust nature of living systems..........9
Population heterogeneity..........11
Exposure measurement error..........12
Questions for further discussion..........14
Conclusions..........16
Acknowledgments..........17
Declaration of interest..........17
References..........17

## Introduction

The process of conducting risk assessments for both physical and chemical agents has evolved over the past century and has increasingly become more formalized (McClellan, 1999; Albert, 1994). The United States Environmental Protection Agency (US EPA), in carrying out its statutory responsibilities, has probably had greater impact than any other federal agency in fostering the use of risk assessment. The National Research Council (NRC, 1983) has offered advice, documented in numerous NRC reports, on the risk assessment process and how it should be used. The process is generally recognized now as having four key components that are traceable to the NRC (1983) report, “Risk Assessment in the Federal Government: Managing the Process": (1) hazard identification; (2) exposure (dose)-response characterization; (3) exposure assessment; and (4) risk characterization. A later NRC (1996) report, “Science and Judgment in Risk Assessment,” proposed that a feedback loop from identified uncertainties that might be reduced through further research be recognized as a fifth step in the process.

The present paper addresses the important second component of the risk assessment process, the exposure (dose)-response characterization for an agent that has been characterized as hazardous. It is obvious that the results of the fourth step noted above, risk characterization, are dependent upon “exposure characterization,” either as measured or estimated for a particular situation, and the nature of the “exposure (dose)-response” relationship.

The published literature and, indeed, most of the NRC reports on risk assessment, frequently refer to dose-response relationships or curves; linear dose-response relationships; linear, nonthreshold dose-response relationships; or nonlinear, threshold dose-response relationships or curves, all making use of the word “dose” in a wide variety of situations. In most cases, what is being described is better termed “exposure,” because uptake and target-tissue levels are seldom directly addressed, and we will use this terminology in the present discussion. Moreover, the term “response” can be ambiguous, and we shall take it to mean the greater or lesser likelihood that a discrete case of the health effect is induced in an exposed subject by the exposure to the agent in question, with the question of how such responses relate to underlying continuous changes in causal biochemical or physiological processes, and how they may relate to the background rate of the same effect in unexposed populations as matters to explore in our discussion.

It is not surprising that, as the need arose for conducting risk assessments on chemical carcinogens, the approach taken was to borrow heavily from what was known about radiation carcinogenesis (Albert, 1994; McClellan, 1999). Indeed, today a cornerstone of assessing the carcinogenic risks of chemicals is that if the chemical or its metabolites cause gene mutations by interacting directly with DNA, they cause cancer in a manner analogous to radiation, and the dose-response relationship for the chemical can be assumed to have a linear, no-threshold relationship. The debate still continues regarding whether this default assumption should or should not be extended to chemicals that are not DNA-reactive and yet cause cancer, typically with protracted high levels of exposure in laboratory rodents.

The debate over the nature of the exposure (dose)-response relationship has nowbeen extended from cancer as the health endpoint to a wide range of noncancer endpoints. These include respiratory and cardiovascular morbidity and mortality. A major stimulus for this debate has been the increased attention given to a group of air pollutants regulated by US EPA under the Clean Air Act as criteria pollutants for which National Ambient Air Quality Standards (NAAQS) must be established. The criteria pollutants include particulate matter (PM), ozone, carbon monoxide, nitrogen oxide, sulfur dioxide, and lead. With sufficiently high levels of exposure, all of these pollutants increase the occurrence of noncancer health effects, such as respiratory diseases, over the background rate. A critical question that arises in developing NAAQS for each of these pollutants is the risk at low levels of ambient exposure.

The debate over the nature of the exposure (dose)-excess effect relationship for noncancer endpoints has been brought to the forefront by two recent publications. A paper by White et al. (2009) describes the conclusions of an ad hoc panel of nine individuals who addressed “issues and approaches in low dose-response extrapolations.” Several of the participants in that endeavor served on the NRC committee that prepared the report, “Science and Decisions: Advancing Risk Assessment” (NRC, 2009). That report proposed three options for extrapolating low-dose risks of both carcinogens and noncarcinogens (conceptual models 1, 2, and 3). Model 1 proposes thresholds for some individuals, but not on a population basis. Model 2 proposes both individual and population thresholds. Model 3 proposes linear and nonthreshold responses for the individual and the population. In practice, one must essentially ascertain that the chemical's effects do not follow any of the mechanisms that are posited as causing linearity to accept it as Model 2. In practice, it is likely that this will almost always lead to the adoption of either Model 1 or 3, both of which assume linearity at the population level.

The question about the appropriate presumptions to make about exposure-response patterns at exposures below those at which frank toxicity is readily observed has a long history of discussion. Many points continue to receive vigorous discussion, as proffered general principles, as bases for science policy decisions and default approaches, and in the risk assessment of particular agents and toxic effects. In the present paper, we focus on one newly salient aspect of this debate—whether agents causing noncancer toxicity at high exposure levels should, as a general principle or a default stance, be presumed to cause some degree of risk of these same endpoints at any positive dose, no matter how low.

Adopting this view for noncancer endpoints could amount to abandoning thresholds as a tenet of biology, pharmacology, and toxicology, and abandoning the no observed adverse effect level (NOAEL)/uncertainty factor (UF) approach to characterizing acceptable levels of exposure to agents causing high-dose toxicity. Although we understand the usefulness for risk management purposes of continuous measures of increasing or decreasing expected impact of an agent on public health as control measures are contemplated, we fear that a hastily adopted approach that assigns some level of risk of induction of full-blown cases of the health effects seen at very high exposures to all lower exposures will be destructive of the credibility of quantitative risk analysis. Moreover, we find such a blanket approach scientifically unjustified, and the balance of this paper presents our reasoning for thinking so.

The arguments proffered as to why all exposure-response relationships should be assumed to be linear include (1) the general “additivity-to-background” argument, which assumes that if an agent enhances an already existing disease-causing process, then even small increases in exposure concentration and/or duration increase disease incidence in a linear manner; (2) the “heterogeneity in the population” argument, which assumes that variations in sensitivity among members of the target population tend to “flatten out and linearize” the exposure-response curve; and (3) a review of epidemiological evidence supposedly showing linear or no-threshold effects at low exposures in humans, despite nonlinear dose-response in the experimental range in animal testing for similar end-points (NRC, 2009; White et al., 2009). (These three arguments were initially laid out by NRC [1977], but that report noted that additional lines of reasoning and additional assumptions need to be recognized in considering the arguments for linearity. Other arguments have been put forth, such as that receptor-mediated endpoints have no threshold. Although we do not agree with this tenet, exploration is beyond the scope of the present discussion.^1^) Below, we raise questions about the transparency, completeness, and scientific validity of each of these arguments for the following reasons: (1) the principle of additivity to background disease processes fails to support linearity unless it is related to a specific mode of action that has some nonuniversal properties—properties that would not be expected for most noncancer effects; (2) although heterogeneity in sensitivity and in modifying factors among people in a human target population may tend to broaden the dose-response relationship, they do not linearize it; and (3) exposure measurement error in epidemiological studies can lead to an apparent linear exposure-response relationship, when the true relationship is nonlinear or threshold.

### Framing the problem

Before beginning our discussion, it is useful to sharpen the question. In our experience, clear and productive discourse on these issues is often hampered by the lack of a common, generally agreed-upon framework and terminology. The low-dose linearity thesis for noncancer toxicity as set out in the “Science and Decisions” report (NRC, 2009) and in White et al. (2009) is put forth in general terms and is taken to be broadly (if not universally) applicable. Their arguments for low-dose linearity are based not on specific observations about specific chemicals and their modes of action, but rather on a proposed broadly operating argument-in-principle—on an assertion that, in their view, some very general and plausibly applicable biological processes underlie apical toxicity generation, which should be expected to lead to small increments in the probability of response at any positive dose level, no matter how small. Assertions are being made about the logical expectations regarding the existence of risks that are well below the observable parts of dose-response relationships. Therefore, in aiming for compelling and broadly applicable analysis, their arguments must be cast in terms of general principles about what ought to be expected regarding the interaction of very low levels of a chemical with the toxicity-generating process. As a consequence of this, our own arguments and critiques must be expressed in similarly general terms.

In this context, the apical effects under discussion are quantal effects—ones that are either present or absent— rather than continuous measures such as blood pressure or liver weight. That is, the “response” in the proposed low-dose-linear dose-response relationship is the probability that an individual would be scored as having developed the apical effect as a consequence of exposure. On a population level, it is the expected fraction of a population that would show the effect. As will be discussed below, however, even though the *apical* endpoints are quantal, their asserted increase in frequency of appearance at very low doses depends on arguments about how those doses quantitatively perturb underlying continuous physiological states, and further, how such small perturbations of underlying continuous variables lead to generation of the quantal apical toxicity of concern. That is, the crux of the matter is the conception of how underlying continuous biological variation—as quantitatively perturbed by small exposures—produces discontinuous transitions from a healthy to a diseased state. Because our subject is the nature of this connection, it is necessary for us to address how small doses affect underlying continuous variables, but the reader should bear in mind that these perturbations are not themselves the apical responses in question, which remain as “cases” of a recognizable and discrete dysfunctional state.

For discrete outcomes, the dose-response curve represents the change in probability that an individual will respond to a given dose by becoming a case. In this context, “probability” refers to the expectation that a randomly chosen individual exposed at a given level will be found to be a responder. In cancer risk assessment, we often treat all individuals as of equal inherent susceptibility, and the probability of responding with a chemically induced tumor is treated as stochastic—a matter of chance that is not probed for the underlying reasons as to why one subject responds and another similarly exposed one does not. That is, the “probability” of apical outcomes enters for each individual as a stochastic phenomenon at the most fundamental and causative level accessible to our conceptional approach. In the present context of noncancer toxicity, however, we are instead dealing with underlying physiological differences among individuals that constitutes the basis of interindividual variations in susceptibility. The overtly observable dose-response pattern is seen as emerging from the interaction of such variation with the doses and with the disease-generating process. In particular cases, one could imagine getting information on this underlying variation in causal processes and using it to make deterministic models of apicaltoxicity generation that results in subject-by-subject determinations of who responds and who does not. If this modeling were perfect, there would be no stochastic element involved—any particular subject's outcome could be calculated from the model—and in a population of randomly chosen individuals, what would vary with dose is the proportions of them listed in the responder and nonresponder columns. When one speaks of “probability” of response in this context, one is referring to the chance that a randomly chosen individual of unknown susceptibility is found to be susceptible enough to be a responder at the dose in question. In practice, one cannot really create an infallible deterministic model, and so the distinction between known-but-varying causes and randomly acting causes breaks down. It is an interesting philosophical debate, beyond the scope of the present paper, to consider whether unknown-but-deterministic causes constitute “chance,” how this may or may not differ from “true stochasticity,” and how the answer should affect the conduct of dose-response analysis. It should be clear, however, that in the present paper, we are addressing the NRC (2009) and White et al. (2009) consideration of hypothetical underlying causes of susceptibility cast in very general terms, critiquing their analysis of how such variation should lead to low-dose linearity. Accordingly, we must do as they have done, playing both sides of the “known or unknown causes” issue to see how they relate. That is, one hypothesizes underlying susceptibility variation and assumes knowledge of it (including variation among individuals) to make arguments about the spectrum of outcomes it should generate; then, one imagines an analyst faced with data on apical outcomes and at most only partial understanding of the particulars of those same toxicity-generating processes, especially on the level of their variation in particular identified individuals. The aim is to make conclusions about how that analyst should analyze the probability of response, including expectations about the probabilities at doses too low to observe outcomes directly. The outcomes are probabilities because the analyst does not share the omniscient point of view about the hypothesized toxicity-generating process.

"Low-dose linearity” of such dose-response relationships, then, refers to the notion that small doses, no matter how small they may be, should be expected to result in some incremental increase in the probability of response (as probability is defined above), over and above any responses that would have happened in any case even in the absence of those small exposures. That is, it is an assertion of the lack of a population exposure threshold for the increase in risk of an induced effect. (As we will discuss below, the argument as presented by NRC [2009] and White et al. [2009] actually appears to require that *individual* thresholds exist—doses below which a given subject will not respond—as a way to generate the discontinuous jump from unaffected to affected at the level of the quantal apical endpoint. But low-dose linearity asserts that there is no *population* threshold, meaning that there will always be some individuals having personal thresholds of zero, and so they will respond to any increment of dose no matter how small.)

As formulated in NRC (2009) and White et al. (2009), the argument for existence in principle should apply equally to animal and human dose-response curves. Nonetheless, if one were to accept this principle and use it in formulating science policy for human health risk assessment, one would need to address the fact that, because the origin of the linearization effect resides in the patterns of interindividual variability and the nature of the background disease processes with which low doses interact, the application of the principle to human health risk estimation would need to rely on inferences and understanding about background disease rates and processes in the target *human* population, and animal dose-response curves, their shapes, and their low-dose extrapolations would have little to say about this subject. Even the animal endpoints would be relevant primarily as providers of hazard information about the interaction of the agent with human background disease processes.

The assertion of low-dose linearity applies to the very bottom of the dose-response curve—that is, that at zero dose, the first derivative of the curve is positive. (Theoretical no-threshold dose-response curves exist that approach zero asymptotically and so have zero slope at zero dose, but the low-dose linearity claim is that small increments of dose over zero immediately produce a linear increase in risk.) The claim is not that this linearity at the lowest doses necessarily results in a continued linear shape at higher doses; that is, it is not necessarily so that an overall linear shape is expected between the very low doses and the beginning of response rates that are observable in animal experiments, much less that linearity in response should be expected among the dose levels observed in experiments. Hence, observed nonlinearity in empirical results does not preclude or refute the possible existence of a low-dose linear component near zero dose. Strict linearity (i.e., lack of curvature, not merely positive slope) prevails only if higher derivatives of the curve near the origin are zero, and they may be nonzero, but even in this case, small increments in dose will produce nearly linear small increments in risk (as can be shown by a Taylor Series expansion) as long as the first derivative is positive (and the limits to how small the dose range needs to be to preserve near-linearity depends on the values of higher-order derivatives).

We mention these technicalities to be clear about what we are discussing, but there is a very practical aspect. Although the discussion in the risk assessment community about the arguments in NRC (2009) and White et al. (2009) have focused on broad principles, the apparent intent of the NRC panel with regard to the practicalities seems to have been lost. The report states (p. 131), “Note that *low dose linear* means that at low doses ‘added risk’ (above background) increases linearly with increasing dose; it does not mean that the dose-response relationship is linear throughout the dose range between zero dose and the high dose.” The associated figure in the report (Figure 5-6) shows clear nonlinearities in the range from the data to zero. We agree with the NRC (2009) authors on this important point. Even if one were to agree with the NRC (2009) assertion of low-dose linearity for classical noncancer endpoints, this does not mean that it is appropriate to estimate risk by drawing a straight line from a point of departure to zero. The question then arises about how one would obtain information regarding the magnitude of the theoretical low-dose linear risk component. The NRC (2009) report provides only minimal ideas in this regard, and it does not address how to extrapolate downward from high doses—that is, how far below the lowest observed doses one might go with a markedly nonlinear extrapolation until one reaches the “low” dose range at which the hypothetical forces causing linearity come to predominate over other influences on curve shape.

Thus, although we agree with the NRC panel that low-dose linearity does not imply linear extrapolation from a toxic effect level, the implications of this statement need further discussion in the risk assessment community. Since the assertion of low-dose linearity of noncancer-effect dose-response curves is about the existence of a linear component at the very lowest doses—and not about its magnitude, about the range of doses over which linearity is expected, or about the shape of the curve at places other than near zero dose—the role of this principle (even if it were to be accepted) in practical noncancer risk assessment is a matter needing much further consideration. Such discussion is beyond the scope of the present paper, but some of the issues have been discussed elsewhere (Rhomberg, 2010). For the present, we wish to address the question of whether low-dose linearity (in the particular sense of the term as we have tried to define it above) should be presumed to exist for quantal noncancer toxicity apical endpoints, either as a general rule applicable to all or most effects, or at least as a likely and frequent effect that is sufficiently prevalent that it might be taken into account in formulating risk assessment science policy.

The further question of whether, as a matter of science policy for risk analysis, human risks at low doses ought to be estimated using some form of linear extrapolation from higher doses—on the grounds that linearity near the origin may exist or at least cannot be dismissed as possible—is a topic for later discussion. But this further discussion cannot be pursued productively until the question of the expected existence of linearity near the origin is better understood, and this narrower question is the focus of the present paper.

## Additivity to background

The general principle of the additivity-to-background argument is that if an agent to any degree enhances the underlying pathobiological process or perturbs the level of some underlying state that is sufficient as a genesis of the background or “spontaneous” cases of disease, then the incremental effect of small increases in exposure to that agent will be a linear increase in response rate over background in the population. The argument was initially proposed by Crump et al. (1976) for application to genotoxic carcinogens. More recently, it has also been suggested to be applicable to common noncancer endpoints, first by Crawford and Wilson (1996), who noted that, if background cases of the endpoint in question (those appearing in unexposed individuals) are considered to be manifestations of the same process that, through its acceleration by exposure, leads to observed toxicity at higher doses, then an argument similar to that put forth by Crump et al. (1976) could apply. Crawford and Wilson (1996) treated the noncancer toxicity as a quantal endpoint appearing with a probability characterized by a dose-response that implicitly uses as its “dose” measure the intensity of the underlying toxicity-generating process. The existence of background cases is then attributed to the native nonzero level of intensity of this process in the absence of exposure to the chemical in question; in other words, the point of zero dose of the agent in question corresponds to a nonzero, positive level of intensity of the underlying toxicity-generating process, and hence the unexposed population is already somewhat “up the curve” from its origin at a level of zero for the ordinate (the probability of response) and zero for the abscissa (the a intensity of the underlying causative process). If one presumes a continuous and monotonically increasing dose-response relationship, the incremental response over background at doses just above zero exposure to the chemical will be approximated by the tangent to the curve at zero dose, which will always be positive and, at least for very small doses, linearly increasing.

Crawford and Wilson (1996), and similar approaches that have followed, presume a connection between underlying biological processes, their perturbation by small increments of exposure, and the probability of generation of a new case of the endpoint in question, but they have not probed its presumed properties. If one considers that noncancer apical toxicity effects are engendered not by rare all-or-none events (such as mutations), but rather as emergent consequences of patterns of changes of sufficient magnitude in continuous physiological variables, through which homeostasis and defensive processes that keep organisms functioning normally even in the face of environmental fluctuation are overcome, then the means for turning small quantitative physiological changes into overt and recognizable cases of frank disease are not necessarily simple. In our view, a presumption that a little more chemical leads to a little more probability of effect is not self evident and needs careful consideration. (Noncancer effects that occur via a mode of action that involves direct DNA interaction may well have exposure-response relationships that are similar in shape to those for classical carcinogens, but most noncancer endpoints result from interaction of the chemical with proteins or other cellular constituents.) Proponents of the additivity-to-background argument for such endpoints propose that heterogeneity in the human population leads some individuals to be at the margins of acceptable levels of such underlying physiological variables, and that the background rates of disease are explicable, at least in part, by the hypothesized existence of individuals who, even without chemical stressors, have values of the underlying variables that are insufficient to maintain health.

The additivity-to-background argument was raised to justify the adoption of a linear-at-low-dose approach as a general principle to be applied to all toxicants and toxicity endpoints regardless of case-specific data (White et al., 2009). Appropriate consideration of this hypothesis requires articulation of the statistical and biological assumptions (many of them implicit) on which this approach is predicated. This includes identification of the assumptions about the nature of the “background processes” and background disease, about the nature of human heterogeneity and its relation to healthy and diseased states, and about the effect of small chemical doses on the biological system.

What makes additivity to background risk plausible for genotoxic carcinogens is the discrete and stochastic nature of both the mutational changes and the malignancy-bearing endpoint of concern—each mutation happens all-or-none and not by degrees. Also important is the single-molecule nature of the component processes, which makes possible the jump from state to state in individual genes borne by individual cells. Finally, events at the molecular level in a single cell can, owing to the proliferation of a resulting malignantly transformed cell through a series of cell divisions, develop into a life-threatening tumor that manifests itself as a fullblown case of disease at the whole-organism level. Thus, there is a process that amplifies the single-molecule, single-cell events into a disease that affects the whole organism. In contrast, for noncancer toxicity, the disease endpoints of interest consist of structural and/or physiological failures of systems at the whole-organism level. Individual molecules or cells are only important insofar as they constitute minute fractions of the overall process acting among many cells and molecules, the sufficiency of the collective function of which is the key to whether disease occurs. The noncancer health effect endpoints themselves vary in severity. Categorizing individuals into those with or without the health effect (and thereby defining the rate of quantal “response” to be characterized by the exposure-response analysis) may involve an arbitrary demarcation between normal and marginal cases. The underlying causal processes are also graded, so there is no discrete jumping from state to state. (There maybe recognizable stages and key events in the progression of the disease process, but they are landmarks in a continuum rather than changes between discrete states such as normal versus mutated.) Despite this underlying continuum of causal processes, we recognize apical toxic effects as marked departures from and alteration of the normal and healthy state. Apical toxicity involves more than being in the tails of distributions of normal states; it entails dysfunction and a cascade of physiological failures with multiplying consequences. Unlike mutations, where the change of state is a single molecular event at a single locus, the generation of noncancer toxicity is a complex process of interacting forces acting throughout the tissue and the organism. This toxicity reflects an emergent property of control networks—or rather of the failure of those control networks—that, instead of dissipating and ameliorating perturbations, come at a certain level to amplify them. We use the concept of a threshold to recognize at a phenomenological level that some values of underlying physiological states maintain normal control and function, whereas others lead to lack of control and dysfunction that is discontinuous with the range of normal variation, even though the magnitude of the excursion from normal states may vary among cases. The challenge for noncancer toxicity dose-response assessment is to account for how the modest degrees of underlying continuous variation are translated into more or less discrete differences between healthy and diseased states.

The logic of additivity to background disease risk is therefore not a direct translation from the genotoxic carcinogen case; one must consider how noncancer effects arise, what accounts for the appearance of background cases, what is changed by the introduction of a toxic agent, and how such an agent is thought to increase the marginal rate of appearance of disease. Despite this lack of equivalence to genotoxic carcinogens, some arguments entailed in how induced cancers might relate to the endogenous disease process are valuable to note and may apply to noncancer effects, albeit in a somewhat altered way: (1) the effect that appears in unexposed individuals (and is being added to) must be the same in underlying pathological process and in ultimate manifestation as the effects induced by exposure; (2) background rates of all needed processes occur even in the absence of exposure; (3) these account for the appearance of the full apical effect as a background rate among the unexposed; (4) even small exposures produce changes in the rates or magnitudes of these processes from what they would be absent such exposure; and (5) the small changes so induced are sufficient to provoke a discrete overall change in health state of the organism from normal or healthy to affected or diseased. In the paragraphs that follow, we examine the logic of additivity to background as it would apply to noncancer toxicity influenced by alteration of underlying continuous physiological states, asking whether the implicit assumptions about the nature of the underlying action of noncancer toxicants and of any consequent adverse effects are indeed in accord with our understanding of toxicological processes for such endpoints.

The notion presented by proponents of additivity to background risk for noncancer toxicity is that there is some key physiological variable or state that varies continuously among individuals. A poor value of this state produces the disease endpoint in question. In essence, there is a threshold value for the internal state, beyond which the frank disease endpoint occurs. The background rate of disease (to which the chemically induced component adds) is attributed to some individuals having a value of the key underlying variable that is beyond the cut-off level for health, and so they have the disease. That is, the background rate of disease occurs because some individuals are beyond the threshold value for the internal factor even in the absence of the toxic agent in question. The agent in question is seen as marginally shifting the distribution of the underlying variable among members of the population such that some individuals who were formerly just marginally adequate for the physiological parameter are now just beyond the threshold and therefore the disease develops. According to this view, then, an amount of exposure, however small, shifts the distribution of the internal physiological state, however slightly, and leads to at least a small number of population members to transition from the healthy to the diseased state. For small increments of exposure, the change in risk should be approximately linear in relationship to increased exposure.

When presented as an argument in principle (rather than as an interpretation of case-specific observations), the identity of the “internal state” or “key physiological variable” is not specified. It is presented as a hypothetical embodiment of the additivity-to-background premise regarding the chemical's ability to enhance or perturb the same processes that lead to background cases of the disease among unexposed individuals. That is, it is something that varies among individuals, for which such variation accounts for the existence of background cases in the absence of exposure, for which small changes are sufficient in at least some individuals to move them from being without the apical endpoint to having the apical endpoint, and for which very small exposures to the chemical will indeed produce those small changes. In evaluating the plausibility and hypothesized generality of this argument—and its comportment with our understanding and observations of underlying biology and noncancer pathogenesis—it is important to identify the specific properties that such underlying variables and endpoint-generating toxicological processes must have for the logic of additivity to background to operate. Moreover, if these principles are to be used to address whether risks to humans at low exposures are to be expected for hazards identified at much higher doses, and in animal tests rather than in humans, then some particular correspondences need to exist between the causal processes at such high doses in animals (where the potential hazard is identified) and very low doses in humans (where the potential for impact of that hazard is being inferred). In particular:

1. Certain particular values of the underlying physiological variable or state must be sufficient to cause the presence or absence of the disease in question. That is, falling beyond a threshold value is enough to cause someone to become a responder. (Otherwise, the crossing of the threshold would not produce an additional case.)
2. There must be a background incidence of disease in the population even without exposure to the causative agent in question, and this must be the same disease state, with the same pathogenesis, as the responses seen with high concentration and/or long duration exposures to the agent in question. That is, the agent must increase the disease rate by shifting the distribution of the underlying physiological variable or state, and crossing the threshold must mean the same thing with the same consequences throughout the dose range and for human and animal. In other words, some fraction of the population is already beyond the threshold value of the internal state, and the physiological or structural failure that constitutes the disease endpoint must therefore be present as a natural and inescapable part of the population variation.
3. Moreover, for application of animal toxicity data to human health risk assessment, the background of human disease (to which the agent is imagined to be adding) must be pathologically equivalent to the induced effect in animals in the sense that development of adverse outcomes is contingent on the same small changes in underlying physiology. (If this is not so, then there is no biologically equivalent human background to add to.)
4. Many of those who do respond with the disease are only slightly different in their key internal state variable than other members of the population who, by being just on the other side of the threshold, are deemed healthy. If the healthy and diseased states for the endpoint in question seem qualitatively distinct, there must be markedly nonlinear processes that, once triggered, cause an individual just marginally different in the underlying causative variable to develop the full disease and become distinct from others with only slightly different values of the internal state.
5. Even small amounts of the agent in question are capable of shifting the distribution of the internal state or physiologic variable. That is, it is assumed that the agent can affect the value of the internal variable without a threshold. Put another way, the body is unable to offer effective resistance to even small further impingements by chemical agents on the internal state variable in question.

As the discussion above makes clear, the added risks being asserted at low exposure levels are a population phenomenon; any particular individual faced with a low exposure will either tolerate it (and remain a non-case) or respond to it (and become a case), depending on how close the preexisting internal state was to the threshold for generating the apical response. Indeed, the additivity-to-background argument relies on the existence of thresholds on the individual to account for the genesis of new cases of the apical effect with only small changes in internal state. (Whether the threshold internal state for a response remains constant for the population and individuals vary in how close to it they are and how much a given dose alters their internal state or whether one considers the threshold to vary among individuals is largely a matter of how one defines the terms; for the generalized case, these are equivalent, but if one is examining actual internal states and values of relevant physiological factors, the distinction becomes important. This issue could arise in applying any presumed general principle to actual disease processes.) Seen this way, it is clear that the core assertion of the additivity-to-background argument (and a possible route to evaluating its biological plausibility) is that the distribution of individual thresholds (or equivalently, the distribution of internal states vis-à-vis a fixed threshold) goes all the way down to zero thresholds and zero tolerance for further alteration for some part of the population. Indeed, the distribution goes even “lower” in the sense that background cases of the endpoint in question, those appearing even in unexposed individuals, are attributed to their having values of internal states and personal thresholds such that, even if one could perturb the internal state a small amount in the direction *opposite* to that produced by the chemical, those individuals would still develop the apical toxicity in question. When the application is to human risk estimation from observed toxicity on animals (as is often the case), there is the further assertion that the pathophysiological processes that led to the toxicity observed in high-dose animals also exist to some degree in all humans, that at least in some humans, they are of sufficient magnitude to generate the same kind of toxicity even without the chemical, and that in others, they are of nearly sufficient magnitude and need but a small increment to lead to the generation of the full apical effect in those people.

Of course, the properties needed for the action of non-cancer agents causing noncancer effects, and for the nature of background incidence of the same endpoints, could be true. But it is important to evaluate the needed properties in view of what we have usually understood about the nature of the disease processes for noncancer endpoints. Below, we raise relevant considerations that lead to the understanding that the needed properties of toxicity-generating processes required for additivity to background for noncancer end-points are not, in fact, generally expected, and indeed are not in accord with our understanding of normal physiology and the degree of its variation among individuals in the human population.

In fact, when we examine noncancer toxicity of agents, including examination of the changes in subjects without the fully developed endpoint in question, we see a progression of stages of increasing impact, and it is not only the final frank effect that shows dependence on exposure level or duration. Pathologists examining such effects recognize a cascading series of ever more consequential failures to maintain normal status in the face of ongoing chemical assaults, but at lower exposure levels, the precursor stages are not merely rarer, they are less severe or (at sufficiently low exposures) entirely absent. It is not until earlier stages become sufficiently advanced and inherent resistance to alteration overcome that subsequent stages are triggered. Moreover, we do not see a continuous gradation in human populations between those few who are fully healthy and those who have varying degrees of advancement of these underlying precursor processes, awaiting only a small chemically induced push to develop into full-blown disease. That is, it is not clear that pathobio-logical progression of states seen to underlie toxic effects in test animals at high doses has a counterpart in humans who are not exposed or exposed only to much smaller amounts. The needed assumptions for additivity to background to operate—that such people “on the verge” of becoming overt cases of adverse outcomes exist, and that their states are continuously graded with a larger population having only slightly better internal states (so that a slightly larger push will push a slightly larger fraction of the population over the threshold)—do not comport with our general observations. Since the gradation of severity of pathology and the need for extreme values of precursor states to generate cases of overt adverse outcomes do indeed appear in animal toxicity studies, the lack of something comparable in the general human population calls into question the needed equivalence of the pathobiological processes to justify the conclusion that small changes in human exposure would generate added cases of the outcome observed at high animal doses.

An additional consideration of the additivity-to-background argument for low-dose linearity relates to the issue of “background” *exposure,* as opposed to background physiological state. This consideration suggests the potential for the “background” exposures of other toxicants to bring the dose-response for an individual chemical (which is biologically nonlinear overall) into the linear range for a human population, as noted by US EPA (2005) and NRC (2008). But this distinction does not affect the principle, since the contribution of background exposures, if they constitute the cause of the background to be added to, must be through effects on the same distribution of the internal physiological variable. That is, the background exposures are simply among the reasons for why the “background” distribution of the internal state is as it is. In any case, as discussed more fully by Dourson and Haber (2010), such “background” exposure to other chemicals is more explicitly addressed as part of a mixtures or “combined exposures” assessment, which is routinely done by scientists based on guidelines from a number of organizations (e.g., ACGIH, 2006; US EPA, 1986, 2000, 2009; IPCS, 2009).

In a credible exposure-response assessment for mixtures or combined exposures drawing maximally on available data based on the toxicity of components as suggested by US EPA (1986, 2000) guidelines and frameworks of international organizations (IPCS, 2009), however, the exposure-response assessment of individual components does not (at least as currently formulated) incorporate considerations of background exposures to other similarly acting chemicals. Rather, a mixtures exposure-response assessment should be conducted on the mixture of concern, or on a sufficiently similar mixture. Absent such information, the mixture dose-response assessment should be based on the dose-response assessments of individual chemicals, and these assessments on individual chemicals cannot in themselves incorporate background exposures to other chemicals.

### The no-threshold proposalfor noncancer toxicity is at variance with decades of experience in observing exposure-response relationships in pharmacology and toxicology, both within and below the usual experimental range for environmental chemicals

The idea that toxic effects have exposure thresholds below which the challenge posed by the agent is insufficient to cause an adverse response is fundamental to the science of toxicology and has been so for decades (Gallo, 2008; Rodricks et al., 2007; Eaton and Gilbert, 2008; Aldridge, 1986; Gehring et al., 1978; Clark, 1937). It is the same first principle that forms the basis for pharmacology and serves as a basis for the use of pharmaceuticals in medical therapy (Dorato et al., 2007; Parascandola, 1981). This enormous weight of experience and observation cannot be overturned lightly. It is not simply that doses without added incidence of frank diseases are commonly observed; it is also that pathological investigation of changes at the underlying molecular, cellular, and physiological levels show that the impacts of chemical exposure are progressively attenuated at lower exposures until exposure levels are reached at which no structural or functional abnormalities are seen. That is, the effects not only become less common with progressively lower doses, they also become less severe, or even adaptive, until a level of interaction of a substance with a biological system is reached that has no ability to perturb the tissues in question from their normal function. This is not a matter of mere assertion on principle; it is based on massive amounts of repeatable observation (Calabrese and Baldwin, 2003; Calabrese and Blain, 2005; Cohen, 2002).

The no-threshold idea is also belied by our experience with medicines, poisons, foodstuffs, and many other kinds of exposure to agents that can have toxic effects if experienced in excess. With the possible exception of allergic reactions, within the range of low exposures, we do not observe slightly increased exposures to such agents somewhat increasing the probability that we will suffer the full effect of a toxic dose. In therapeutics, a small fraction of the therapeutic dose will not necessarily produce a moderate or full response in a diminished fraction of the treated population. It is only when the critical concentration is sustained at the site of action for the necessary period of time that an effect will be elicited. The experience of exposure thresholds for biological effects, including adverse effects, pervades daily life.

Indeed, the very notions of “toxicity” and “pathology” would not exist if there were basic and gradual continuity in the properties of tissues and physiology between a healthy state and a diseased one. Conversely, the very notion of “normal” and “healthy” would not exist if it were impossible to recognize a qualitative (and not merely a graded) distinction between affected and unaffected tissues. In short, the notion that the underlying determinants of and progression toward frank overt disease vary continuously in the whole population, that the background incidence of frank disease reflects only being in the tail of such a distribution, and that such individuals differ only in degree from a substantial number of others who have an underlying pathology not yet severe enough to count as cases of disease is not in accord with our general experience or our observation of the underlying states of tissues and physiology.

This is most evident with severe acute toxic effects. For example, acute arsenic poisoning can be lethal (Levin-Scherz et al., 1987; Campbell and Alvarez, 1989; Quatrehomme et al., 1992; ATSDR, 2007). The amounts required to produce such effects are much greater than usual human exposures, not only in populations exposed to low arsenic levels (< 1 ppb in drinking water), but also in populations exposed to high arsenic levels (>1000 ppb) (Petito-Boyce etal., 2008). Although these populations differ significantly in development of chronic effects of arsenic, acute lethal toxicity requires large amounts more of arsenic in either population.

Indeed, if it were so (as additivity to background demands) that there are members of the population just on the verge of thresholds for the tolerable state of internal variables, and that small exposures can shift those variables, then there should be no distinction between acute and chronic toxicity. For a hypothetical person on the verge of the threshold, even a small one-time exposure might be all it takes to move the internal state just enough to cross the threshold, and so toxicities that appear in some people only after prolonged, ongoing, and substantial exposure should appear in those on the verge of response after only momentary small exposures. The fact that we do indeed recognize distinct modes of acute and chronic toxicity, and that agents cause different outcomes from short and prolonged exposures, argues against the generality of the needed assumptions for additivity to background (Rodricks et al., 2007).

### The no-threshold proposal is at variance with basic tenets ofhomeostasis—the robust nature of living systems

NRC (2009) suggested that threshold-based tolerance to a chemical may not exist in individuals who have reduced or no capacity to deal with that chemical. This is often described as infinite sensitivity (i.e., there is no dose so small that it will not affect some person in the population) and has been suggested to be caused by other stressors (e.g., background exposure to chemicals, physical stressors, presence of disease) that diminish capacity in individuals or genetic factors that result in a subpopulation of already ill individuals that respond to any added exposure.

Organisms are continually faced with changing circumstances and surroundings, including variations in diet and exposure to environmental substances. The internal milieu must be kept in a very different state (in terms of moisture, pH, chemical concentrations, DNA integrity, and a host of other factors) than that prevailing in the external world, and this difference must be maintained by the constant expenditure of energy lest the forces of thermodynamic equilibration destroy the special structures, compositions, and interactions that constitute the living state (NRC, 2007a). At the level of cells and organelles, it is the maintenance of different states inside and outside, as well as from cell to cell, that give them their functional properties; an orderly, purposeful control of such states and their transitions to alternative settings as needed is essential, and the ability to maintain that order is paramount (Goodman et al., 2010).

This includes, but goes beyond, homeostasis and feedback mechanisms. As systems-biology approaches to understanding organisms’ functioning become more prominent in our investigations of physiology, its modulation and perturbation, it becomes ever clearer that complex interacting gene expression networks embody stabilizing functions that lessen the impact of fluctuations and steer the overall state toward one of a few rather discrete states, with switching from one stable state to another effected by pronounced and carefully modulated signals (Ideker et al., 2001; Lusis, 2006; Conolly and Thomas, 2007). It is typical for component processes to be stimulated by one type of molecule and inhibited or undone by another, with the overall state being maintained by feedback processes that tune the outcomes to desired levels.

It is a fundamental property of living systems that they have extensive means to buffer their internal physiological state against the effects of changes the environment might tend to have on them (e.g., the ability to deal with free radicals, reactive oxygen species, and reactive products produced in the normal metabolism of food; Kemper et al., 2007). Indeed, the ability to control and appropriately modulate the internal physiological state, and not to have that state be buffeted about by changes in the external environment or elsewhere in the organism, is often named as one of the defining properties of life (Sadava et al., 2009). It is this process that ceases at death, and this cessation is quickly followed by deterioration and decay as the thermodynamic equilibrium formerly held at bay takes its course.

This robustness of the living system in the face of environmental perturbations—this ability to carry on life processes in an appropriate and orderly way despite the day-to-day and moment-to-moment fluctuations, novelties, trends, and vicissitudes of impinging forces—comes to bear on the threshold question in two ways. First, the robustness is part of the organism's “defenses” that the threshold model deems in need of being overwhelmed if a dose is to be large enough to cause toxicity (Sielken and Stevenson, 1998; Rhomberg, 2004). The defenses are not merely detoxification and sequestration of the agent; they also include the resistance of the biological system to being unduly changed by the agent's presence. It is when the ability of the system to counter that impingement becomes exhausted that the changes of control, and thus of functioning, become evident, and the dysfunction and destruction characteristic of toxicity begins. Thus, the existence of the robustness mechanism embodies the basis of the exposure threshold, and the mode of its failure defines the nature of the toxicity that ensues.

Strictly speaking, the additivity-to-background argument does not deny that this kind of systemic stability and homeostasis operates and indeed that it constitutes the way in which those individuals who are not especially sensitive manage to tolerate exposures to the chemical in question. The assertion is that individuals vary in these properties sufficiently that, at least in some of them, the resistance to environmental perturbation is so minimal that even small exposures to a chemical overcome the defenses. The counter to this line of argument is that we do not in fact observe this degree of variation—we do not see continuous and gradual gradation between people with normally functioning physiology (with some range of variation, but always adequate to maintaining basic functioning) and those who cannot maintain internal states in the face of environmental fluctuation or cannot coordinate their cells to function as tissues or cannot carry out basic life processes. It is not that individuals who have such problems do not exist; it is rather that such individuals are recognized as having a distinct state of disease, with specific causes of those diseases, and there is not a gradual gradation of such people with the general population such that health and disease are but extremes on a continuum.

The second way that robustness comes to bear on thresholds is that its existence contradicts a needed assumption for the additivity-to-background argument: namely, the notion that small exposures to an agent will cause corresponding shifts in the distribution of some key internal physiological variable, pushing some individuals who are just on the margin of an acceptable value over the limit to a value that engenders toxicity. In view of robustness, it is not clear that small doses will indeed shift such a distribution. Although the value of the key internal variable may vary among individuals, for any one individual it will tend to be controlled by that person's set of feedback and homeostatic processes, and accordingly it will be resistant to change from that value. That is, the additivity-to-background argument, when applied to noncancer toxicity, requires that the agent act on the value of the underlying physiological variable without a threshold. But in fact, as with the apical toxicity endpoint, the component physiological processes have robustness to small forces acting on them, and they will require a certain amount of force to overcome this resistance to change (Stebbing, 2003, 2009; Rodricks et al., 2007). (It has also been argued that some responses can be hormetic, meaning there is a statistically or biologically significant decrease in adverse effects below the background effect in the low-dose region. A hormetic response in any low dose-response assessment would necessitate a threshold for an adverse effect at higher doses, and would obviate a linear low-dose approach [Dourson and Haber, 2010].)

The additivity-to-background argument fails to support general linearity. To conclude that the end result of a biological process is linear, one must start by assuming that the underlying processes sufficient for (and not merely risk factors of) disease are linear; thus, one would be assuming what one is trying to prove. This is obviously circular reasoning. One must also assume that an agent will cause an effect, at even the smallest dose, and this assumes that there are no homeostatic or defensive mechanisms to regulate the biological process in question. This is tantamount to assuming that one-one millionth of a gram of aspirin would be sufficient to cause metabolic acidosis or gastrointestinal (GI) hemorrhage in at least some exposed people. Although low-dose linearity could occur under some circumstances, the assumptions would be very particular and do not likely apply in most circumstances.

In the end, the simple view of noncancer toxicity as expressed in the additivity-to-background argument—that toxicity hinges on the sliding value of a single underlying key physiological variable—oversimplifies the question. A real understanding of noncancer dose-response entails embracing the complexity of underlying processes. Toxicity (whether cancer or other endpoints) is a multistep process. One molecular or cellular event or slightly altered physiological variable does not result in a toxicity endpoint being manifested (Rhomberg, 2004). Multiple molecular and cellular changes and events, each with their own threshold and dose-response, are necessary to produce the cancer or noncancer effect. In addition, the role of repair and detoxification in toxic process must be considered. Finally, effects are temporal in nature and are also sequential, in that actual modes of toxic action entail initial perturbations, adaptive responses to those perturbations (designed to minimize alteration of physiological control), and initial failures at lower levels of organization, which only if prolonged lead to a further cascade of failures that ultimately manifest themselves as sufficient overall dysfunction to be considered an adverse apical endpoint.

## Population heterogeneity

**Figure 1:**
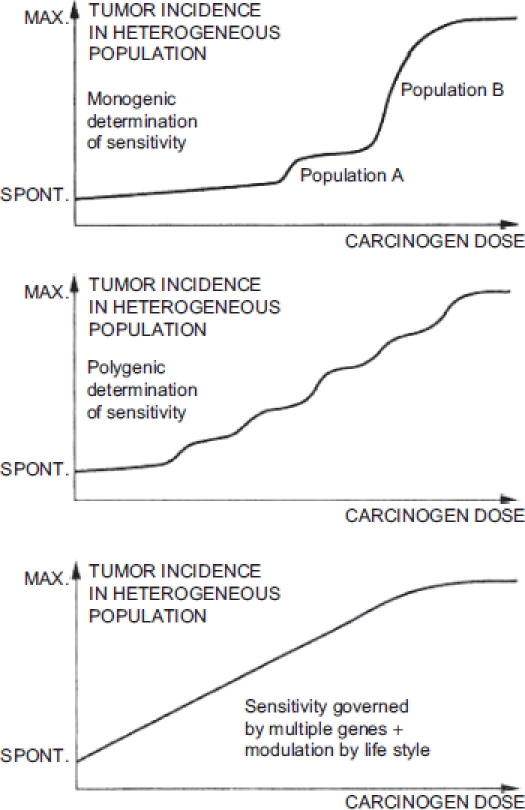
Schematic representation presented by Lutz (1990) of doseresponse relationships in heterogeneous populations, with increasing factors affecting sensitivity to a chemical carcinogen in each graph.

White et al. (2009) asserted that heterogeneity in sensitivity to environmental chemicals in the population tends to “linearize” the dose-response curve. They suggested that a linear function for the population will result even if dose-response associations are not linear in individuals. They referred to the argument put forth by Lutz (1990 94-3541) regarding cancer and suggested that this argument holds for noncancer effects as well. Lutz (1990, p. 1246) claimed that because “sensitivity is governed by a number of genes, the dose-response curve becomes flatter with each modulatory factor (Figure 4 [reproduced here as Figure 1]). When the number of factors is increased *ad infinitum,* linearity results between the spontaneous tumor incidence and the high dose incidence. Although this situation cannot be reached in reality, the linearizing effect of population heterogeneity might be sufficiently strong to account for the fact that dose-response curves in most human epidemiological studies do not show significant deviation from linearity."

This argument does not actually hold for carcinogenesis, much less noncancer health effects. Lutz (1990) presented a widely cited diagram to illustrate his assertion, but it is only an illustration and is not (nor was it intended to be) the outcome of an analysis or the application of an established principle. Yet there is a mathematical principle applicable to this situation: using Lutz's line of reasoning leads to the conclusion that heterogeneity in population sensitivity leads to a lognormal, not a linear, curve.

There are two issues with applying Lutz's reasoning to the shape of the dose-response curve. The first is structural— under the tolerance-distribution approach, dose-response is *itself an* expression of heterogeneous susceptibility. That is, dose-response curves showthe distribution among members of the population in their individual level of sensitivity to a chemical; at higher and higher doses, a smaller and smaller proportion of the population can tolerate the exposure without ill effect (because their personal thresholds have not been exceeded). When dose-response patterns are observed in data, they are direct manifestations of the variation in sensitivity and the operation in the population of factors altering that sensitivity. In other words, in experimental investigations of toxicity, variations in the individuals responding at different doses provide data on population variation in sensitivity. Accordingly, it is not clear what it would mean to have sources of variation in the sensitivity to an agent as external factors to be superimposed on an observed dose-response. How are those factors to be viewed as additions to or outside influences on the operation of the set of sensitivity variations that led to there being an observed dose-response relationship in the first place?

The second issue is that the combined action of multiple sensitivity factors is not to linearize the dose-response curve, but to make it lognormal. The best way to regard the action of sensitivity factors is as multiplicative modifiers. For example, if a difference in uptake of a chemical alters a biological event in a disease process 3-fold and differences in metabolism alter it 5-fold, then combined, this would lead to a 15-fold difference in biological events leading to disease. Each factor magnifies or lessens the rates of processes responsible for tolerance or susceptibility. The combined effect of many independent multiplicative factors, according to the central limit theorem, is to produce a lognormal distribution of sensitivity. As stated by Morgan etal. (1990, p. 89), “The lognormal distribution applies as the limiting case for multiplicative quantities due to the approach to normality of the sum of the logs."

The applicability of this theory was demonstrated by Hattis et al. (2001), who created a database of quantitative observations of parameters likely to affect responses to particles. This study was based on epidemiological studies of methacholine, flour dust, and other agents that induce acute changes in lung function. These investigators found that lognormal distributions provided a good description of their vast data set. Although the slopes of the lines describing the data covered a considerable range, perhaps reflective of measurement errors or differences in populations in the different studies, these investigators reported an absence of any systematic departures from the expected lognormal distributions in the data sets, and supported the use of a simple distributional model for risk projections.

Based on basic principles and actual data, it is evident that heterogeneity in the population regarding sensitivities to chemicals will not lead to a linear dose-response curve. Rather, the multiplicative effects of the factors that affect sensitivity result in a lognormal curve.

## Exposure measurement error

White et al. (2009) cited some epidemiological studies that appear to show linear/no-threshold exposure-response patterns even when corresponding studies conducted in laboratory animals, using relatively similar exposure regimens, suggest otherwise. They interpreted these results as demonstrating the linearization of exposure-response in heterogeneous human populations; that is, the observation served for them as a confirmation in observational outcomes of their proposal that additivity to background and population heterogeneity effectively negate any threshold or nonlinear pattern that might be inherent in the toxicity mode of action. Further, in responding to a critique of White et al. (2009), Burke et al. (2009) stated, “Although [a small range of exposures and measurement error] need to be considered in evaluating epidemiologic study results, modeling techniques such as nonparametric smoothing methods have demonstrated the capacity to identify potential threshold relationships even in the context of relatively extreme measurement error (Cakmak *etal.* 1999; Schwartz and Zanobetti 2000)."

It is well recognized in the regression literature, however, that measurement error in the independent variable generally biases the observed functional relationship with the dependent variable toward a more modest and flatter trend than is actually the case (e.g., Carroll et al., 2006; Kuha and Temple, 2003; Crump, 2005; Küchenhoff and Carroll, 1997). The measurement error need not be biased itself; the simple fact that the placement of individual observations along the *x*-axis is “smeared out” (since the measured values place them randomly higher or lower on the axis than their true position, owing to the imprecision of the measurements) tends to obscure any inflections in the functional relationship and makes it appear more linear than it truly would be seen to be if the dependent variable were measured without such errors. It is important to recognize that the issue here is not simply that studies may have poor statistical power to show that nonlinearities are significantly supported over alternative linear model fits (although that issue also exists), but rather that exposure measurement error can artificially give the appearance of linearity to an exposure-response relationship that is truly sharply nonlinear or even threshold in nature.

In epidemiological studies of exposure-response patterns, the independent variable is the exposure measure, and measurement uncertainty in individual exposures is typically pronounced. This is especially so for criteria air pollutants, such as PM, for which exposures for nearby individuals may be estimated using results at centrally located air-monitoring stations. Exposure measurement error can result when concentrations of the pollutant measured at central monitors are not representative of personal exposures to the pollutant. Reasons for this include uneven distribution of PM attributable to local sources; monitoring sites may represent a nearby source and not human exposures a small distance away; pollution patterns can be affected by terrain features and weather; and daily variations in PM concentrations at a central monitoring site may differ from variations experienced by individuals (US EPA, 2009). Even when personal exposures can be measured more directly, they typically represent “snapshot” determinations at a particular moment in time, and each such single measurement is an uncertain indicator of the long-term past cumulative or average exposures for the individual in question, yet it is these long-term past exposures that are at issue for many toxic responses that are being evaluated for a population of individuals. Moreover, differences from person to person in the relationship between externally measured exposure and the internal, target-organ biologically effective dose will lead to further exposure measurement imprecision. Even in the unlikely event in which all individuals in a population have the same true concentration-response threshold, exposure measurement error could result in some individuals appearing to be affected below this threshold and others appearing not to be affected even above their true threshold. Thus, thresholds in the relationship between individual exposures and risks of morbidity and mortality may be blurred by measurement error when studied by using the relationships between concentrations at central-site monitors and aggregate morbidity or mortality (Daniels et al., 2004).

**Figure 2:**
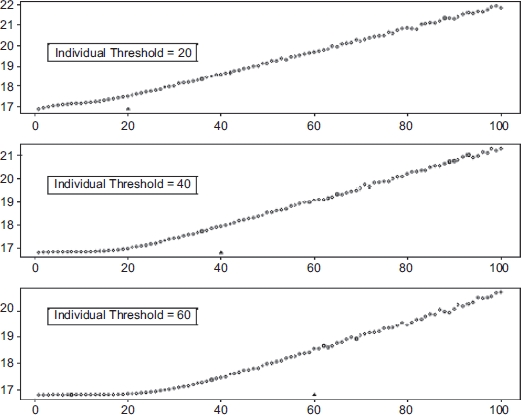
Simulation results reproduced from Brauer et al. (2002). The graphs show ambient concentration of PM_25_ (μg/m^3^) on the *x*-axis and the expected number of deaths per 1, 000, 000 on the *y*-axis. The true underlying individual thresholds are indicated on the graphs as triangles.

Because of the prevalence of exposure measurement error in epidemiological data, conclusions about the linearity of the exposure-response curve must be examined carefully and treated with some skepticism. The study that best illustrates the effects of measurement error on assessing thresholds was that by Brauer et al. (2002), who conducted simulations in which a strict population threshold at various levels of exposure was assumed, and then the effect of measurement error on the ability to detect that threshold, or even a nonlinear exposure-response pattern, was assessed (Figure 2). It is noteworthy that Brauer et al. (2002) estimated the magnitude of exposure measurement error (which was assumed to be unbiased) based on actual studies of criteria pollutants (measured ambient and personal PM_2_._5_ and sulfate concentrations)— that is, the magnitude of uncertainty in individual exposure measurements was drawn from actual cases of agents for which apparently linear dose-response patterns have been claimed. These investigators found that estimating individual exposure to air pollutants from central-site outdoor pollution monitors may result in considerable error. They reported that some individuals in the population will have greater exposures than others for any given central-site ambient concentration, which will broaden the normal distribution of risks due to interindividual variability in exposure.

The Brauer et al. (2002) simulations set the true exposure-response curve (i.e., true personal exposure versus response) by assumption, using strict thresholds shared by all members of the population that were set at various exposure levels together with a linear rise in risk above this threshold. They then simulated the exposure-response relationship that would be found by a study in which each true curve was affected by exposure measurement error of the magnitude actually observed to occur in real studies. They showed that if exposure measurement error could be reduced by the use of appropriate exposure metrics, then common underlying individual thresholds result in similar population-level thresholds. But at the levels of exposure measurement error actually found in criteria pollutant epidemiological studies, the simulated exposure-response curves looked linear even when the specified true curves had thresholds. (See Figure 2, drawn from their paper.) It was further shown that the obscuring of thresholds would be greater if the simulations incorporated thresholds that varied among individuals (yet for which there was nonetheless a strict population threshold below which no person responds). The simulations of Brauer et al. (2002) suggested that the inability to detect a threshold in many epidemiological studies does not, in fact, mean that no threshold exists. The apparent linearity of observed exposure-response relationships in studies with prevailing levels of exposure measurement error is explicable as an artifact that arises from the biasing effect of exposure measurement imprecision.

It has been suggested that robust epidemiological investigations have shown that thresholds are not generally observed for noncancer outcomes, citing studies of radiation, secondhand tobacco smoke, nitrogen and sulfur oxides, PM, ozone, and lead (White et al., 2009). To use PM as an example, in its most recent Integrated Science Assessment, US EPA (2009) evaluated newly available evidence to assess a population threshold value for health effects. They cited studies by Daniels et al. (2004), Schwartz (2004), and Samoli et al. (2005), which assessed associations between short-term PM_2_ _5_ exposure and mortality rates, and the Schwartz et al. (2008) study, which assessed association between long-term PM_2_ _5_ exposure and mortality rates. US EPA (2009) concluded that these studies consistently found that a no-threshold log-linear model adequately portrays the PM concentration excess mortality response relationship in multicity analyses, although uncertainty exists on a city-to-city basis because of heterogeneity in the exposure concentration-response curve across cities.

Burke et al. (2009) cited the Cakmak et al. (1999) and Schwartz and Zanobetti (2000) studies as evidence of modeling techniques that can identify potential thresholds in exposure-excess risk relationships. Although Schwartz and Zanobetti (2000) argued that it is possible to detect threshold relationships in meta-analyses down to low concentration levels, they did not actually directly address the effect of measurement error on the shape of the concentration excess response within individual cities upon which the meta-analyses were based. Cakmak et al. (1999) evaluated whether nonparametric smoothed representations of the association between air pollution and mortality could distinguish between linear and threshold models in the presence of measurement error. The investigators dealt only with population-level data, however, and did not directly address the impact of individual measurement error on the shape of the concentration-response curve. In addition, they showed that with simulations of threshold models, although a threshold model was selected over a linear model the majority of the time, this “majority” ranged from 52% to 90%, depending on the exposure error and threshold concentration in the simulated data. Thus, between 10% and 48% of the time, a linear model was incorrectly chosen over a threshold model.

Although exposure measurement error may not always be large enough to appear to linearize a truly threshold dose-response relationship, it is important to bear in mind that this bias exists and that, in simulations, the degree of bias known to apply to actual studies is sufficient to produce a false, apparently linear exposure-excess risk result. That is, it is not just an issue of statistical power to detect nonlinearity, but rather that exposure measurement error biases towards finding flatter, more linear exposure-response relationships than the actual underlying truth. This has not only been shown by Brauer et al. (2002), but is a generally recognized statistical principle that uncertainty in an independent variable generally flattens a regression curve (Zeger et al., 2000). Because exposure is the independent variable in an exposure-response relationship, measurement error leads to uncertainty, which then leads to a flattening of the curve.

Even if one were to adequately address issues of exposure measurement error, there could still be other methodological issues that could affect the interpretation of an exposure-response analysis. For example, confounders (e.g., co-exposures to other agents, diet, genetics, socioeconomic factors, or lifestyle) may not adequately be accounted for or disease ascertainment may not be robust. These factors could impact the exposure-response model chosen.

Many factors can mask a threshold for exposure-excess disease risk in human studies, so it is critical that mode of action for the agent in question be considered. If a threshold is not detected in a human study, but there is a scientifically valid reason based on the mode of action for the agent that the effect is of a threshold nature, then one must conclude that apparent differences in the concentration-response function shape between human data and animal studies relates not to actual linearity in humans (as White et al. [2009] argue), but rather to this artifactual flattening, which can be detected by observing the mode of action and its nonlinearities in animal experiments, where exposure measurement error can be minimized.

## Questions for further discussion

The abandonment of a threshold approach to noncancer risk assessment is an enormous step, and it is important that the underlying assumptions and implications of any new methodology be fully understood before rash alterations are made. The questions about what to expect from low exposures for noncancer endpoints for noncancer toxicity, and how variation in the population and heterogeneity in sensitivity impact the potential impact of additivity to background and other matters, are not simple. We have attempted to more explicitly address underlying assumptions and relevant available data as a basis to consider more fully low-exposure linearity in further dialogue and exploration.

The larger problem we face in noncancer human health risk assessment is that we seek insight into the potential for lowlevels of exposure to cause harm to humans, and we must use as a basis for such judgments the observations from hazards evident at much higher exposures, often in animals. The human risks at low doses, if they exist, are too rare to observe directly, and so inferences must be made that depend for their validity on invoking wider biological understanding of what should be expected to occur at low levels of human exposure. Our main need is to extrapolate downward from observed effects at high doses, using the presumption that the dose-response pattern seen in animals is giving us useful information about the human dose-response pattern and its lower dose range.

In view of this, it is important that the additivity-to-background argument—the lynchpin of the proposal to apply low-dose linearity to noncancer risk assessment—is an assertion of the existence of a linear component to the population dose-response curve at the very lowest doses.

That is, it is about extrapolating *upward* from the zero-dose point rather than *downward* from observable responses or points of departure. Even if one were to accept that such an effect happens at near-zero exposures (and we have taken issue above with the main arguments that suggest this is generally so), it remains that the existence-in-principle near zero dose has little to say about the magnitude of the risk or how far upward from zero dose one can go before any theoretical linear component is overwhelmed by the nonlinear aspects of the mode of action of most noncancer endpoints. Because the reason for the existence of such a near-zero linear component has to do with the pattern of variation in sensitivity in the subject population, it is human background diseases and human variations in the values of underlying contributing factors that are at issue, and animal studies provide little information about these. The dose-response pattern observable in animals depends on interindividual variation in animals rather than in humans, and moreover in the central and least-sensitive part of the animal variation range, not the most-sensitive end that is at issue for additivity to background. In our evaluation of the linearity question, we have focused on a critical look at the arguments for the existence of a low-dose linear component, but even if such a component is accepted, many further questions will need to be examined if one is to know how to make use of this principle to address how to conduct top-down, animal-to-human dose-response inferences, and a number of consequences, many apparently unintended, would arise if linear extrapolation from points of departure were adopted as a science policy (Rhomberg, 2010).

There are some particular larger questions that we think deserve deeper examination and wider discussion. One is the issue of “harmonization” of cancer and noncancer exposure-response approaches for risk assessment purposes (Bogdanffy etal., 2001; Barton etal., 1998; Rhomberg, 2004). Although one can hardly be against harmony and for discord, it is important to realize (taking the metaphor of “harmony” as a model) that harmonization does not mean that an identical method needs to be applied to all cases for all agents and disease endpoints. Indeed, in the musical definition of harmony, the point is that all the notes are *not* identical; different notes complement one another and are compatible. The combination of different, yet harmonized, notes allows for a greater richness in the music by drawing on a larger harmonic framework with which each note is compatible but no note is identical. “Harmonization” should mean stressing compatibility of inferences about underlying pharmacokinetics and modes of action and using the insights developed in dissecting the underlying biology of different endpoints to inform our understanding across endpoints.

Clearly, in selecting exposure-response methods, we should aim at approaches that are not contradictory or incompatible, but this does not mean that they should be identical. There are notable differences between some cancer and noncancer responses that lead to different primary reasons why a changing response rate with increasing dose is observed (Rhomberg, 2004). In cancer, the issue has traditionally been the greater probability of the confluence of a number of all-or-none events, each of which happens with a dose-dependent probability. Response is largely a matter of chance as to which individual cells achieve the requisite set of genetic changes, and the modeling of such chance events (among individuals who are not otherwise notably different) is often assumed to be the driver of the dose-response phenomenon. It is now recognized, however, that some carcinogenic events—such as mutation, homeostasis, DNA repair, and checkpoint control— may in fact have thresholds. This is recognized in US EPA's cancer guidelines (US EPA, 2005), which suggest a nonlinear approach to cancer risk assessment when carcinogenesis occurs via a mode of action that is not linear at low doses. For typical noncancer endpoints, in contrast, the effect is on the collective function of all the molecular pathways, cells, and tissues that are involved in the relevant physiological processes and the endpoint. Toxicity manifest as noncancer endpoints essentially consists of the consequences of dysfunction of the whole physiological system, not of failures of any particular cell or gene. The component physiological processes vary continuously, according to the sum of the functioning of all the cells and molecules in the tissues in question in processes that are characterized by homeostasis and active controls to maintain and properly modulate desirable states in the face of environmental stresses. Toxicity consists of the failures of overall control, and stages of the process can be recognized, from adaptive and compensatory processes that are the signs of the successful operation of the control processes through adverse dysfunction and finally, to frank toxicity. It is a basic characteristic of living things that they maintain controlled internal states in the face of external fluctuations in their environments, fluctuations that include uptake of small amounts of substances from the external world. The nature of biochemical machinery, with extensive feedback and control processes and means for orderly and purposive changes in state, is geared to providing the means for organisms not to be buffeted about into adverse states by small environmental stressors, and a good proportion of energy use by living systems is devoted to maintaining such controlled states that differ from and retain independence of the surrounding environment. Toxicity should be seen as the failure of such processes, and the effect of low stressor levels needs to be tied to the nature of those failures.

The recent NRC report, “Science and Decisions: Advancing Risk Assessment” (NRC, 2009), noted that cancer exposure-response is driven by a stochastic-event approach but does not incorporate information on individual sensitivity, whereas the noncancer toxicity exposure-response approach is driven by considerations of individual sensitivity differences but does not treat stochastic elements. The NRC report advocated incorporating both approaches into all exposure-response assessments in the interest of harmonization and consistency. Although this seems like a good idea in principle, the underlying assumptions need to be stated and further explored. Moreover, the practicality is that exposure-response data from experimental studies typically have few exposure groups and a premium already exists on the ability to estimate a sufficient number of parameters by fitting models to data; the current dichotomy of approaches toward cancer and noncancer endpoints reflects the attempt to devote the few dose-response curve parameters that may be set by fitting to data to the characterization of the main processes that are felt to be the dominant determinants of the modulation of response level with differing exposures. As a basis to meaningfully develop a harmonized approach further, the nature of appropriate data to best inform suggested approaches will need to be considered. A simple multiplying of distributions seems unwarranted—if there is indeed interindividual variation in sensitivity to the causation of component events, then the variations in the events themselves are what need to be characterized.

Another related question is to examine what it means to separate consideration of sensitivity from dose-response for a noncancer endpoint. As noted above, variation in sensitivity has, in existing practice, been considered as the reason for the observed dose-response—that is, as the basis for the increasing proportion of responders at higher doses. Considering dose-response for an individual of specific sensitivity invokes an alternative determinant of response. Stochastic event models of noncancer toxicity have not been developed, and it is not immediately clear how such models would relate to the biological causes of the events they aim to describe.

As the issue of harmonization of risk assessment approaches is debated, it is important to ask what is the primary objective of the risk assessment endeavor. The authors of this paper are evaluating the potential overall health impact of exposure to multiple agents that may produce multiple endpoints. The impact of these different endpoints (cancers and the different noncancer effects for individuals and populations) are not the same. Thus, it does not make sense to use the same approach to model the effects.

Finally, we must return to the motivations for the proposal to undertake linear low-exposure extrapolation as a general default procedure. As we have stated, we do not think such an approach is scientifically justified or supported. One motivation is to base characterization of potential impacts of small exposures on a precautionary basis, raising theoretical possibilities that can be abandoned if sufficient proof against them is adduced. We caution that, aside from the lack of scientific justification for such an approach, it is not at all clear that adoption of universal low-dose linear assessment approaches is indeed precautionary. The arguments adduced for such linearity are arguments in principle—they do not provide the basis for estimating the magnitude of any such effect, and it is not clear how to do so in a meaningful way. Moreover, since all substances show toxicity at sufficiently high doses, it would follow from the all-is-linear approach that all exposures to all agents bear some risks. It is not at all clear that action against one hypothetical risk (by restricting exposure on a precautionary basis) would not increase overall risk by inducing added risks from the inevitable increases in other exposures to other agents that any restriction on a single substance would entail. Since the magnitude of the hypothesized risks cannot readily be estimated, one has a hard time assuring that actions against single exposures produce an overall public health benefit.

Another motivation is to enable risk management evaluations that adjustlevels of exposure restriction by the degree of public health benefits they produce. To assess the benefits of regulation or to judge how trade-offs are best made requires a measure of continuous change in marginal impact with marginal changes in exposure—i.e., at least local approximate linearity. We note, however, that such analysis is only useful if it applies to the range of cases being evaluated, and it need not apply universally. We suggest developing approaches that examine exposure-response relationships at the upper reaches—near the clear effect levels—and to do so case by case, bringing understanding of chemical-specific modes of action to bear, to address such questions, rather than to invoke a universal principle of questionable scientific soundness and severely limited practical utility to the question that motivated it.

## Conclusions

Recently, a proposal has been put forth that additivity to background disease and heterogeneity in sensitivity to chemicals in the population is best characterized by linear exposure-response relationships for noncancer health effects at low exposures, and that this is demonstrated in epidemiological studies that show no evidence of a threshold for noncancer health effects (White et al., 2009; NRC, 2009). Assumptions that form the basis for the hypotheses of additivity to background disease and heterogeneity and epidemiological studies that appear to show linear no-threshold exposure-response relationships have been critically examined as a basis for more robust consideration of the proposal. Specifically, fundamental biological and biochemical processes—such as homeostasis, nonlinear signal processing, and the law of mass action— influence and govern exposure-dependent responses to both endogenous and exogenous substances, and to both “toxic” and “therapeutic” agents, and need to be carefully considered and appropriately weighted against potentially policy-based supposition. The development of advanced therapeutics has resulted from increased knowledge of molecular and biological processes brought about by advances in chemistry and biology. Similarly, advances in the use of toxicological and epidemiological data in risk assessment should be driven by evidence-based knowledge of fundamental biological and chemical principles. As discussed here, the default assumption of low-exposure linear extrapolation of toxicity observed at high exposures in all cases does not comport to modern-day scientific knowledge of biology and in the absence of compelling science-based justification.

For both cancer and noncancer responses, default risk assessment procedures are decades old and poorly reflective of current knowledge of biology or of adaptive cellular pathways, particularly those related to the evolution of stress response pathways that protect all cells and organisms from modest increases in concentrations of compounds with potentially adverse consequences at high exposure levels (Simmons et al., 2009). NRC published two documents in 2007, “Toxicity Testing in the 21st Century: A Vision and a Strategy” and “Applications of Toxicogenomic Technologies to Predictive Toxicology and Risk,” both of which argued that new testing approaches need to be developed to evaluate perturbations of human toxicity pathways and to evaluate when perturbations are likely to become sufficiently large to pose a health risk. These documents put forth that all toxicity pathways (including cancer) have multiple stages that are defined events, and that nonlinearity or thresholds in any step will introduce nonlinearity and thresholds in all subsequent steps, including the apical effect. It also asserted that, even if each step is linear, extreme nonlinearity in the dose-response relationship for later steps and apical endpoints will result if there are no background frequencies of individuals at the penultimate stage. In addition, the increasing use of genomic tools is tending to show that thresholds for key events beyond general stress happen at doses close to the doses at which histological effects are observed (even for carcinogens), and at lower doses, several new studies suggest changes are general stress changes, whereas there are no changes in gene regulation (e.g., see Naciff et al., 2005). At later stages closer to the apical effects, more systems in the body (and hence higher levels of organization) are affected. Thus, later stages could have more interactions with disease processes, chemical-disease interactions would not be expected in the early stages of the toxicity pathway, and thresholds in processes in stages prior to the stages in common with disease processes will result in thresholds for that chemical. Based on these principles, one must conclude that thresholds are the rule (NRC, 2007a, 2007b).
